# Phenotypic and genetic analysis of energy partitioning and feed efficiency in Atlantic salmon

**DOI:** 10.1186/s12711-026-01045-w

**Published:** 2026-05-08

**Authors:** Anna Kristina Sonesson, Gareth Frank Difford, Ashie Norris, Bjarne Hatlen

**Affiliations:** 1 Fisheries and Aquaculture Research, Nofima, Norwegian Institute for Food, Post Box 210, NO-1431 Ås, Norway; 2 Department of Animal and Aquacultural Sciences, Norwegian University of Life Sciences, P.O. Box 5003, 1432 Ås, Norway; 3 Mowi Genetics AS, Sandviksboder 77A, NO-5035 Bergen, Norway; 4 Fisheries and Aquaculture Research, Nofima, Norwegian Institute for Food, Sjølsengvegen 22, NO-6600 Sunndalsøra, Norway

## Abstract

**Background:**

There are both economic and environmental motivations to improve feed efficiency. However, direct genetic improvement of feed efficiency ratio (FER; weight gain/feed intake) is difficult. Alternatively, improved FER might be achieved indirectly by selection for increased growth rate and reduced body fat. The aims of this study were (i) to perform a phenotypic analysis of energy partitioning traits among Atlantic salmon families; (ii) to estimate heritability and genetic relationships among feed efficiency and underlying traits; (iii) to determine an optimal breeding strategy to improve feed efficiency in Atlantic salmon.

**Results:**

Moderate genomic heritability estimates were obtained for most traits, e.g. feed intake (0.35), weight gain (0.42), feed efficiency ratio (0.19) and energy density of the gained weight (0.26). Heritability for residual feed intake was 0.04. Genetic correlation between feed efficiency and growth or energy density of the gained weight was 0.68 and -0.64, respectively. A selection index including weight gain and energy density of the gained weight was most beneficial to increase FER. The effect of body energy (reflecting fat deposition) was half of what could be predicted if energy efficiency (EE; energy gain/energy intake) was constant. The fish retained 50 and 49% of the energy and protein intake, respectively. Energy loss was due to heat (29% of intake), faecal loss (18%) and nitrogen excretion (3%). The derived energy and protein budget traits displayed low to moderate genomic heritability (h2 = 0.01–0.31). Protein efficiency reflected FER.

**Conclusions:**

Genetic selection for weight gain and against energy density of the gained weight will improve feed efficiency ratio in Atlantic salmon without the need for individual feed intake records. The results suggest that by selection against body energy, and given the same weight gain, 50% of the reduction in body energy can be realised as reduced feed intake and thus improved FER.

## Background

Feed accounts for around half of the production costs [1] and 85% of the greenhouse gas emissions of Norwegian Atlantic salmon at harvest [2]. Not surprisingly there are considerable economic and environmental motivations to improve feed efficiency.

Feed efficiency ratio (*FER*; weight gain/feed intake) or its inverse feed conversion ratio (*FCR*) varies between salmon families [3, 4]. Direct selection for increased feed efficiency is difficult because it requires accurate measurements of feed intake in large numbers of fish, and finding practically feasible, affordable ways to measure long term feed intake in individual fish with sufficient accuracy has proven to be an intractable problem [5, 6]. Whilst feed efficiency has been genetically improved indirectly in farmed salmon through a correlated response to selection for increased growth [7], direct selection would in principle be more efficient.

Since the relationship between feed intake and feed efficiency is curvilinear [8], the correlated response between growth rate and feed efficiency is expected to decrease with improved growth and reach a point of diminishing returns. Therefore, alternative selection index traits are needed to improve feed efficiency by selective breeding. Given that growth rate is the acquisition of body mass, and the composition of that mass can vary greatly, further investigation into partitioning phenotypes is needed.

FER can be related to the energetic components of feed utilization and expressed as:

1 $$FER=EF*\frac{EE}{EG},$$

where *EF* is energy density in the feed, *EE* is energy efficiency (energy gain/energy intake) and *EG* is the energy density of the gained weight. For a whole growth cycle, *EG* will equal the final body energy density of the fish. Thus, with a given feed composition, *FER* can be improved either by improved energy efficiency or by reducing the energy density of the fish. While *EE* is even more difficult to record than *FER*, body energy is strongly correlated to fat content and both are relatively easy to analyse by standard laboratory methods. Further, selection of live breeding candidates based on body energy may be within reach, as several non-destructive methods hold promise to estimate fat in whole fish, although these have so far mainly been calibrated against muscle fat, e.g. [9–13]. Body fat may, therefore, be an alternative selection criterion trait to select against to increase feed efficiency in fish without the need for records on feed intake as suggested by Knap and Kause [14].

Two fish with identical weight gain can have very different body fat composition and different fat depots across the fillet, viscera and remaining carcass, and in the case of the latter two this constitutes a high-cost slaughter waste [15]. Reduced body fat may possibly be associated with higher energy losses through metabolism and excretion, and thus lower *EE* instead of improving *FER*. It has been shown that direct selection for increased weight gain and reduced muscle fat, was effective in improving feed efficiency in rainbow trout [16] and European whitefish [17]. However, so far, no analysis has been done to estimate the correlated response between body energy and *FER* in Atlantic salmon. Whole slaughter sized Atlantic salmon from Norwegian farms have been reported to contain 21–23% crude fat, accounting for around 2/3 of the body energy [18, 19], suggesting a considerable potential to increase feed efficiency by reducing the total body fat content. However, body fat cannot be reduced below acceptable consumer levels or levels with negative effect on fish health.

The aims of this study were (i) to perform a phenotypic analysis of energy partitioning and budget traits among Atlantic salmon families; (ii) to estimate heritability and genetic relationships among feed efficiency and its underlying traits; (iii) to determine an optimal breeding strategy to improve feed efficiency.

## Methods

### Fish experiment

Individually tagged (PIT-tags, Biomark Inc., Idaho, USA) and genotyped Atlantic salmon from 35 full-sib families of MOWIs breeding nucleus population were transported from Mowi Genetics AS (Øyerhamn, Norway) to the Nofima Research Station for Aquaculture (Sunndalsøra, Norway) as parr of 40 g average body weight. After one month of acclimation in 35 small tanks (one family per tank), a pooled sample of eight randomly selected fish from each family was collected for initial chemical analyses. In addition, 50 randomly selected fish were individually weighed (W0) and distributed into two 150-L experimental tanks per family, with 25 fish assigned to each tank. The 70 experimental tanks were in two different rooms (*ROOM)* with 20 and 50 tanks, respectively. All tanks were supplied with fresh water of stable temperature measured daily in two of the tanks; it varied between 11.9 and 12.3 °C (average 12.1 °C) over the experimental period of seven weeks. The fish were kept under continuous light over the entire period.

The fish were fed in excess with water-stable 3 mm feed pellets produced by extrusion at Nofima’s Feed Technology Centre (Bergen, Norway). The feed was formulated according to commercial practices and yttrium oxide (Y_2_O_3_) was added as an inert digestibility marker (Table 1). The feed was analysed by methods described below, and the analysed composition is given in Table 1. All spill feed (i.e. uneaten feed) was collected from the water outlet and quantified daily after correction for recovery and dry matter content [20]. A recovery test was done in each of the tanks without fish before the start of the trial, using the same conditions (water flow, temperature, etc.) as in the experiment. Of the 1750 fish used in the experiment, two fish were lost. At the end of the trial, faeces were collected by stripping [21], pooled per tank and stored at −20 °C until analysed. The fish were then returned to the tanks and fasted for two days to empty the gut before being euthanized, individually weighed (final weight, *W1*) and stored at −20 °C.

**Table 1:** Formulation of the experimental diet

Formulation	% inclusion
Fish meal	30
Soy protein concentrate	14
Wheat gluten	13
Corn gluten	5
Wheat	15.7
Fish oil	9
Rapeseed oil	8
Micronutrients and additives	5.29
Yttrium oxide	0.01
*Proximate composition*	
Dry matter	92.1%
Crude protein	45.7%
Crude fat	21.0%
Ash	6.9%
Yttrium	82 mg/kg
Energy	22.5 MJ/kg

At the final sampling a random sample of 60 fish of the total of 1748 fish were used for the study of the individual relationship between their whole-body moisture, crude fat, crude protein (nitrogen * 6.25) and energy, using analytical methods described below.

The remaining fish (N = 1688) were pooled into one sample for each of the 70 tanks (21–25 fish per sample). The pooled whole-body samples were homogenized and analysed for nitrogen and energy, as described below, to obtain final tank and family mean values.

Data on initial body nitrogen (*BN0*, see below) and energy content (*BE0*, see below) was missing from one family.

### Chemical analyses of feed, faeces and fish

Whole-body samples were minced, and a representative aliquot homogenised in liquid nitrogen to obtain a homogeneous sample. Before analyses, whole body and faeces samples were freeze dried and moisture measured as drying loss. The feed was analysed for moisture (drying loss at 103 °C to stable weight; ISO 6496: 1999) and ash (combustion at 550 °C, ISO 5984: 2002). Crude fat was analysed in the feed (Soxhlet, after acid hydrolysis) and in the 60 individual fish (Soxhlet). Energy was analysed in all samples (feed, faeces, the pooled-per-tank fish samples and the 60 individual fish)) using bomb calorimetry (ISO 9831:1998). Nitrogen was also analysed in all samples by the Kjeldahl method and crude protein (*CP*) calculated as N * 6.25. Because of limited amounts of faeces, either energy (2 tanks) or nitrogen analysis (3 tanks), or both (2 tanks), were omitted for some tanks. Yttrium in feed and faeces was analysed by inductively coupled plasma mass spectroscopy (ICP-MS).

### Traits

The traits, except those that were recorded on the 60 fish at the final sampling, were all expressed as tank means for the 25 fish in each tank.

Weight gain, *WTGAIN* (g) = *W1*-*W0*, where *W1* and *W0* are final and initial body weight, respectively.

Feed intake, *FI* (g/fish), as the sum of daily feed intake recorded during the entire experimental period.

Feed efficiency ratio, *FER* = *WTGAIN*/*FI.*

Energy density of gained weight, *EG* (kJ/g) = (*W1* * *BE1*- *W0* * *BE0*)/*WTGAIN,* where *BE1* and *BE0* are final and initial body energy concentrations (kJ/g), respectively.

In addition, we calculated and studied 13 derived traits in the energy and nitrogen budgets. These traits were calculated based on metabolic body weight using geometric mean body weight and metabolic weight coefficients of 0.8 for energy and 0.7 for protein. Residual feed intake was calculated as *RFI* = Observed *FI*—Estimated *FI* where the last term was obtained from Eq. (2).

Energy intake, *EI* (kJ kg^−0.8^d^−1^) = *FI* * *E*_*feed*_ /*W*_*geom*_^0.8^/d, where *E*_*feed*_ (kJ/g) is the energy concentration in the feed, *W*_*geom*_, (kg) = (W0 * W1)^1/2^ is the geometric mean weight, and d is number of days between the initial and final weighing.

Nitrogen intake, *NI* (mg kg^−0.7^d^−1^) = 10 * *FI* * *N*_*feed*_ /*W*_*geom*_^0.7^/d, where *N*_*feed*_ (%) is the nitrogen concentration in the feed.

Faecal energy loss (kJ kg^−0.8^d^−1^), *FE* = *EI* * (*Y*_*feed*_ /*Y*_*faeces*_) × (*E*_*faeces*_ /*E*_*feed*_), where *Y* is yttrium (inert digestibility marker) concentration and *E*_*faeces*_ and *E*_*feed*_ are the energy in faeces and feed concentration, respectively

Faecal nitrogen loss (mg kg^−0.7^d^−1^), *FN* = *NI* * (*Y*_*feed*_ /*Y*_*faeces*_) * (*N*_*faeces*_ /*N*_*feed*_), where *N* is the nitrogen concentration in faeces and feed

Energy gain, *EGAIN* (kJ kg^−0.8^d^−1^) = (*W1* * *BE1*- *W0* * *BE0*)/ *W*_*geom*_^0.8^/d, where *BE1* and *BE0* are final and initial body energy concentrations (kJ/g), respectively

Nitrogen gain, *NGAIN* (mg kg^−0.7^ d^−1^) = 10 * (*W1* * *BN1*—*W0* * *BN0*) /*W*_*geom*_^0.7^/*d*, where *BN1* and *BN0* are final and initial body nitrogen concentration (%), respectively,

Branchial and urinary nitrogen excretion (mg kg^−0.7^ d^−1^), *BUN* = *NI*-*NGAIN-FN*

Energy loss in branchial and urinary nitrogen excretory products, *EBUN* (kJ kg^−0.8^d^−1^), was calculated using enthalpy of combustion values of 24.9 kJ g^−1^ nitrogen and 23.1 kJ g^−1^ nitrogen for ammonia and urea, respectively [22], and assuming 15% of the nitrogen excreted as urea and 85% as ammonia [23]; *EBUN* = *BUN* (mg/fish) * 24.63 (kJ/g) /*W*_*geom*_^0.8^/*d*, where 24.63 is the weighted enthalpy of combustion (0.85 * 24.9 + 0.15 * 23.1 = 24.63 kJ g⁻^1^ N).

Heat loss, *HEAT* (kJ kg^−0.8^d.^−1^) = *EI*- (*EGAIN* + *EBUN* + *FE*)

Energy efficiency (%), *EE* = 100 * *EGAIN*/*EI*

Protein efficiency (%), *PE* = 100 * *NGAIN*/*NI*

Digestible energy efficiency (%), *DEE* = 100 * *EGAIN*/(*EI*-*FE*)

Digestible protein efficiency (%), *DPE* = 100 * *NGAIN*/(*NI*-*FN*)

### Genotyping

Genomic DNA was extracted from fin clips of the surviving 1748 fish using a commercial kit (DNeasy Blood & Tissue Kit, Qiagen), following the manufacturer’s instructions. The fish were individually genotyped by a customised single nucleotide polymorphisms (SNP) Affymetrix array for Atlantic salmon developed by MOWI. Genotypic data was filtered using the Plink software excluding SNPs with minor allele frequency (MAF) lower than 5%, missing more than 15% of the SNP genotypes, and/or with one or more Mendelian inheritance errors. 87 SNPs were excluded, and the used numbers of SNPs was 55,648.

### Statistical analyses

For the 60 fish analyzed individually at the final sampling, a linear regression analysis was performed of whole-body moisture, crude protein and energy on crude fat.

### Estimating the effect of different energy sinks (W0, WTGAIN, EG) and FAMILY on FI

The effect of *W0, WTGAIN*, *EG* and *FAMILY* on *FI* was analysed using a linear mixed model applied to the tank means data:

2 $$ \begin{aligned} FI_{{rtf}} = & ROOM_{r} + \beta _{1} W0_{t} + \beta _{2} WTGAIN_{t} \\ & + \beta _{3} EG_{t} + FAMILY_{f} + Error_{t} , \\ \end{aligned} $$

where *ROOM*_*r*_ is a fixed effect of the room (*r* = 1 or 2) in which the tanks were located; *FAMILY*_*f*_ is a random effect of the fullsib family (*f* = 1, 2, …34), each reared in two replicated tanks (*t* = 1, 2, … 68 (two tanks omitted due to missing faecal data); *β*_*1*_, *β*_*2*_ and *β*_*3*_ are the partial regression coefficients of *FI* on *W0*, *WTGAIN* and *EG*, respectively; and *Error*_*t*_ is the random residual of tank *t*.

Residual feed intake (*RFI, g)* was calculated as the difference between the observed *FI* and the estimated *FI* from Eq. (2).

### Estimates of genetic parameters

Variance components of the random SNP effects for each of the derived traits in the energy and protein budget, and for RFI, were obtained from the following linear model:

3 $$ y = {\mathbf{X}}*{\mathbf{ROOM}} + {\mathbf{Z}}*{\mathbf{s}} + {\mathbf{e}}, $$

where ***y*** is a (68 × 1) vector of the tank means for the actual trait; **ROOM** is a (2 × 1) vector of room effects as defined in Eq. (2) and **X** is its (68 × 2) design matrix; **s** is a vector of the random SNP effects for the trait; **Z** is a (68 × *N*_*snps*_) vector of the average SNP genotypes of the fish in each tank, where SNP genotypes are coded as$\left( {C_{ij} - {2}p_{j} } \right)/\sqrt {\left( {{2}\sum {p_{j} \left( {{1} - p_{j} } \right)} } \right)}$ , where *C*_*ij*_ is the average allele count of the reference allele of *j*-th SNP genotype in tank *i*, *p*_*j*_ is the allele frequency for SNP *j*; ***e*** is a (68 × 1) vector of residuals for each of the tanks.

Estimates of (co)variances of the random SNP effects for the four traits *FI*, *WTGAIN*, *FER* and *EG* were obtained from a multitrait model using the ASReml software [24]. The multitrait model was:

4 $$ {\mathbf{y}} = \left( {{\mathbf{X}} \otimes {\boldsymbol{I}}_{4} } \right) {\mathbf{ROOM}} + \left( {{\mathbf{Z}} \otimes {\boldsymbol{I}}_{4} } \right){\mathbf{s}} + {\mathbf{e}} $$

where **y** is a ((4*68) × 1) vector of tank means; **ROOM** is a (8 × 1) vector of room*trait effects; **I**_**4**_ is a 4 × 4 diagonal identity matrix. The variance of the trait-effects of SNP *j* is Var(**s**_**j**_) = **G**, where **G** is the (4 × 4) genetic (co)variance matrix of the traits. Similarly, Var(**e**_**t**_) = **R** where **R** is the (4 × 4) residual covariance matrix of the traits in tank *t*. Since the tank means for each trait are averaged over the 25 fish in each tank, the actual residual (co)variances are a factor 25 larger than **R**, i.e. 25*R [25]. The latter assumes that there are no common environment effects due to the tanks. The effects of this assumption will be reviewed in the Discussion section.

### Alternative selection indices and their resulting multitrait genetic gains

Alternative selection indices were compared to improve the two assumed breeding objective traits *WTGAIN* and *FI* with four different sets of one or two of the selection criterion traits *WTGAIN*, *FI*, *EG* and *FER*. Three relative weights of the two breeding objective traits were expressed per genetic standard deviation of each trait; + 1.0 unit profit increase for *WTGAIN* and -0.25, -0.5 or -1.0 unit feed cost for *FI*.

The selection index **I**_**i**_ = **b’y**_**i**_ of individual *i* is obtained as the linear function of its trait values, **y**_**i**_, with the index weights **b** = **P**^−1^ **G v**, where **P** is the phenotypic (co)variances among the selection criteria, **G** is the genetic (co)variance matrix among the selection criteria traits and the two breeding objective traits*,* and ***v*** is the vector of weights. The vector of genetic gains per trait was calculated as

5 $$\Delta \mathbf{G}=\overline{i}\frac{{\mathbf{b} }^{\mathbf{^{\prime}}}\mathbf{G}}{\sqrt{{\mathbf{b}}^{\mathbf{^{\prime}}}\mathbf{P}\mathbf{b}}},$$

where $\overline{i }$ is the selection intensity. Four scenarios with different selection indices were compared. In scenario 1 with *WTGAIN* as the only selection criterion, while an additional selection criterion was added in scenarios 2 (*FI*), 3 (*EG*) and 4 (*FER*), respectively. The (co)variances of **P** and **G** were those obtained from this study (Table 5). The selection indices contained only their own performance information (**y**_**i**_) for the four traits. We assume that additional information on e.g. sibs would not noticeably affect the direction of the selection response of the traits among the four different scenarios.

With the positive weight given to *WTGAIN* and the three sets of negative weight(s) to FI we expected an increased (favorable) genetic change in *WTGAIN* and a decreased (favorable) genetic change in *FI*, while both the sign and magnitude of the correlated genetic gains in *FER* and *EG* depend on the weights given to the two breeding objective traits and on the genetic correlations among all four traits.

## Results

### Phenotypic analysis

**Fig. 1:**
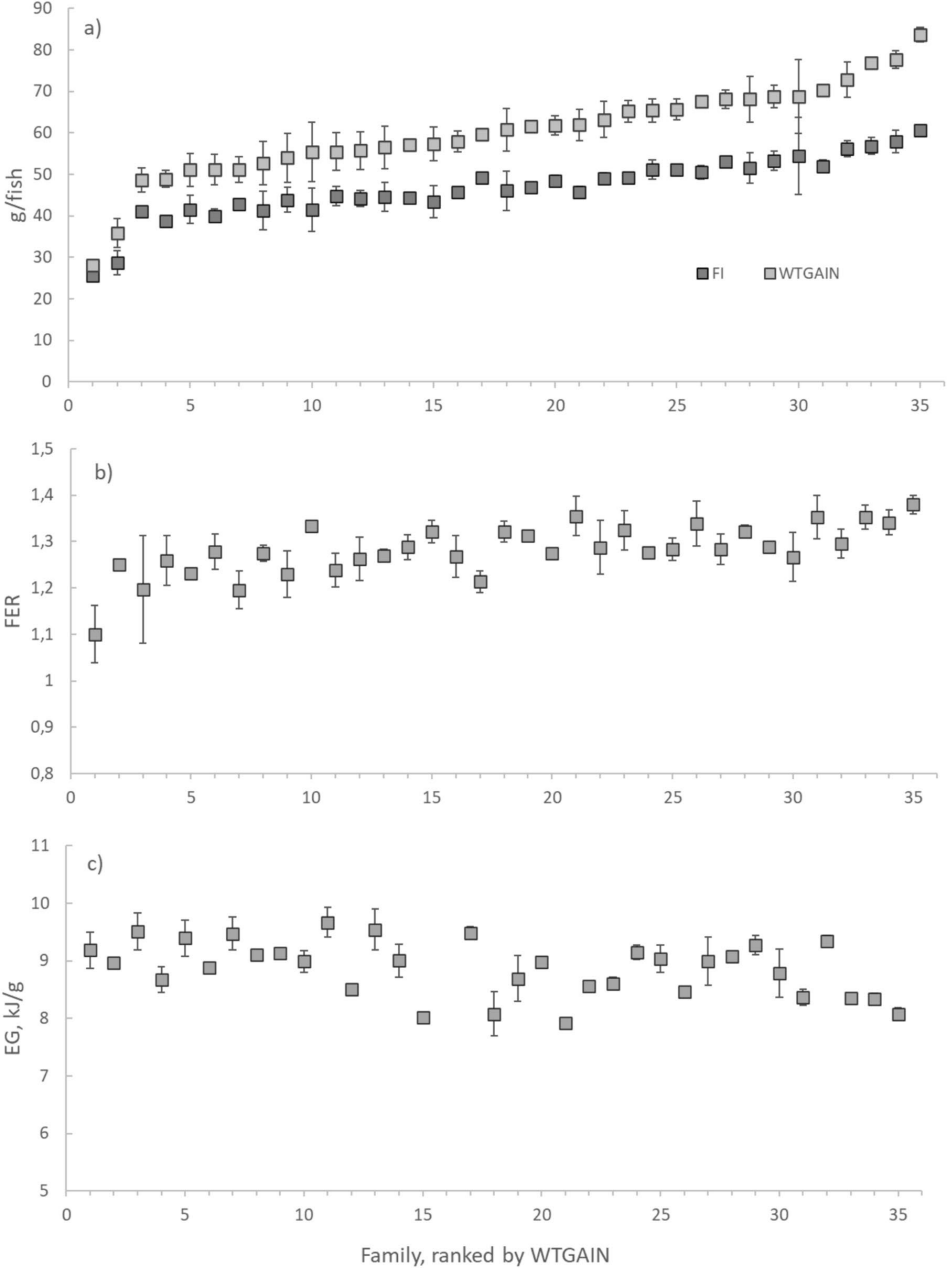
Family means for growth and feed traits ranked by weight gain. Panels show **a** feed intake (FI; g/fish) and weight gain (WTGAIN; g; 35 families), **b** feed efficiency ratio (FER; 35 families), **c** energy density of gained weight (EG; kJ/g; 34 families). Results are means of two tanks/family ± s.d

The 35 families grew from a start weight of on average 50 (*sd* 5) g to a final weight of on average 110 (*sd* 15) g. The weight gain (*WTGAIN*) per family varied from 28 to 84 g (mean 60.1, *sd* 11.0 g) while feed intake (*FI*) per family varied from 26 to 61 g (mean 46.7, *sd* 7.3 g; Fig. 1a). The feed efficiency ratio (*FER*) per family varied from 1.10 to 1.38 (mean 1.28, *sd* 0.005), i.e. a difference of 27% (Fig. 1b). The energy density of gained weight (*EG)* per family varied from 7.9 to 9.7 kJ/g (mean 8.87, *sd* 0.48).

**Fig. 2:**
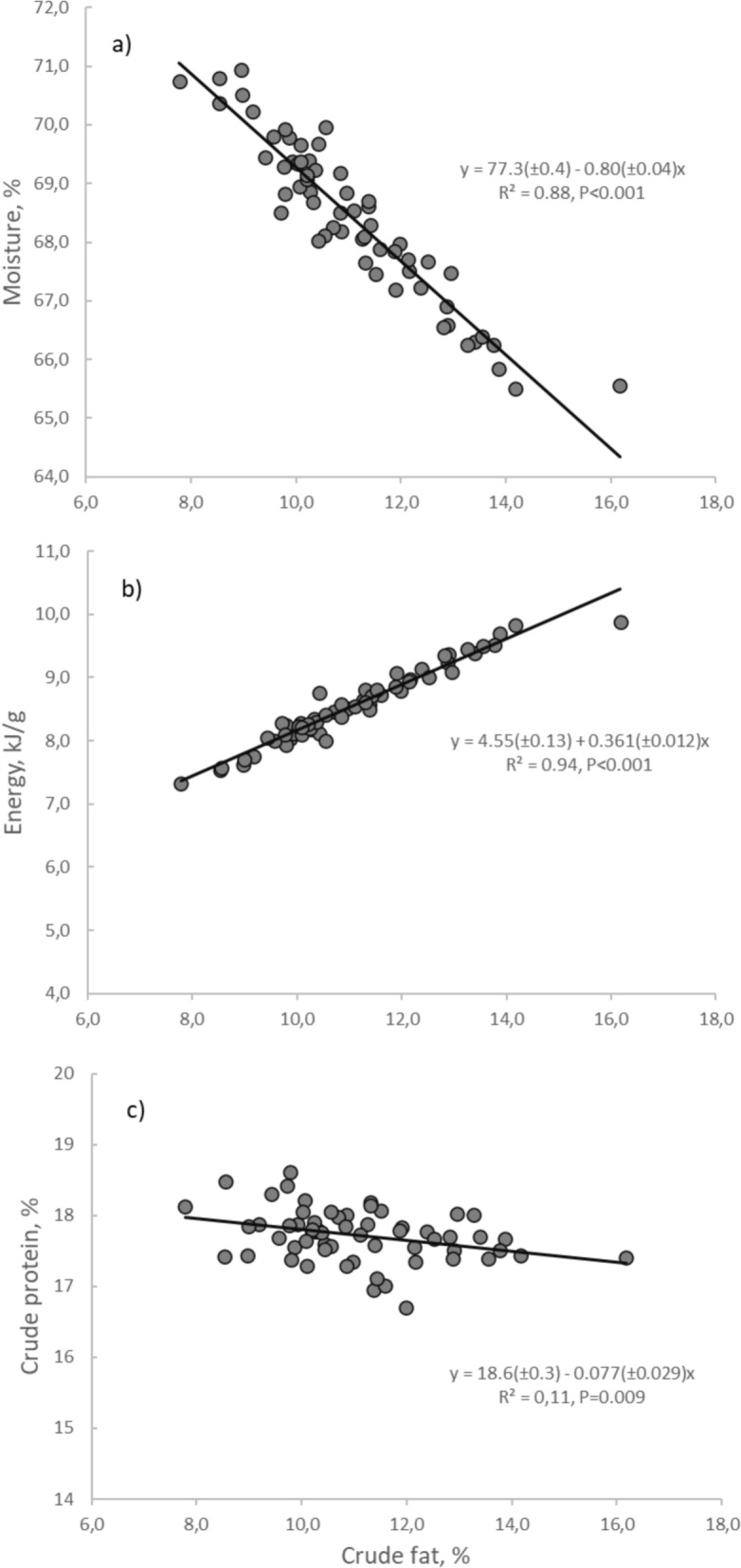
Regression of body composition traits on body crude fat in Atlantic salmon. Each point represents the value for an individual fish (N = 60). Panels show **a** moisture (%), **b** energy (kJ/g), **c** crude protein (%)

For the individual data of the 60 randomly selected fish, body fat was strongly negatively correlated with body moisture (*R*^*2*^ = 0.88, Fig. 2a), strongly positively correlated to body energy (*R*^*2*^ = 0.94, Fig. 2b), and weakly negatively correlated with body protein (*R*^*2*^ = 0.010, Fig. 2c). The regression coefficient of body energy (kJ/g) on body crude fat (%) was 0.361. For a fish of 100 g, 1 g increase in body crude fat (i.e. 1% increase), was associated with a 36.1 kJ increase in body energy (100 * 0.361 kJ/g).

Descriptive statistics of family means of the traits in the energy and protein budgets are shown in Tables 2 and 3, respectively. With respect to the traits in the energy budget, the family mean for *EE* was 50.0 (*sd* = 2.0) % varying from 45.0 to 53.2 (*CV* = 3%). Most of the energy loss per family was *HEAT* (29% of intake, calculated by difference) or via faeces (*FE*, 18%), whereas the nitrogen excretory products (*EBUN*) only accounted for 3.2% of the total energy intake. The experimental design did not allow partitioning of heat into basal metabolism, heat increment and activity. With respect to traits in the protein budget, the mean protein efficiency (*PE*) was 49 (sd = 2.3) %, with family means ranging from 44.0 to 52.2 (*CV* = 4.5%). Faecal losses of nitrogen (*FN*) were 12% of the intake whereas 40% of the ingested protein was catabolized and excreted (*BUN*, calculated by difference).

**Table 2:** Descriptive statistics and heritability estimates for the traits in the energy budget (34 families, 61 tanks)

Trait	Abbr	Mean	Min	Max	Std. dev	*CV*, %	*h*^*2*^
*kJ kg*^*−0.8*^* d*^*−1*^							
Energy intake	*EI*	165	113	190	14.9	9.0	0.25 ± 0.09
Faecal energy loss	*FE*	31	19	44	4.6	15.0	0.17 ± 0.07
Energy of branchial urinary N excretion	*EBUN*	5	4	7	0.5	9.2	0.01 ± 0.07
Heat loss	*HEAT*	46	33	57	4.9	10.5	0.12 ± 0.06
Energy gain	*EGAIN*	83	52	103	9.3	11.2	0.25 ± 0.09
*% of energy intake*							
Faecal energy loss	–	18	15	24	1.8	9.7	0.07 ± 0.04
Energy of branchial urinary N excretion	–	3.2	2.8	3.6	0.2	5.9	0.27 ± 0.10
Heat loss	–	28	20	35	2.9	10.4	0.11 ± 0.06
Energy gain (= energy efficiency)	*EE*	50	46	55	2.2	4.4	0.12 ± 0.06
Digestible energy efficiency	*DEE*	62	55	70	3.2	5.2	0.18 ± 0.08

One family was omitted because of lacking initial sample. Seven tanks were omitted because of lacking faecal nitrogen and/or energy measurements. Consequently, data are based on 61 tanks including 27 duplicate and seven single tank families

**Table 3:** Descriptive statistics and heritability estimate for the traits in the nitrogen (N) budget (34 families, 63 tanks)

Trait	Abbr	Mean	Min	Max	Std. dev	*CV*, %	*h*^*2*^
*mg kg*^*−0.7*^* d*^*−1*^							
N Intake	*NI*	414	274	480	39.4	9.5	0.26 ± 0.09
Faecal N loss	*FN*	49	30	71	8.4	17.2	0.14 ± 0.07
Branchial urinary N excretion	*BUN*	164	117	207	15.9	9.7	0.13 ± 0.07
N gain	*NGAIN*	202	126	244	23.1	11.4	0.31 ± 0.07
*% of N intake*							
Faecal N loss	–	12	9	16	1.5	13.1	0.09 ± 0.05
Branchial urinary N excretion	–	40	35	45	2.3	5.9	0.24 ± 0.09
N gain (= Protein efficiency)	*PE*	49	41	54	2.3	4.8	0.16 ± 0.07
Digestible protein efficiency	*DPE*	55	48	60	2.5	4.5	0.16 ± 0.06

One family was omitted because of lacking initial sample. Five tanks were omitted because of lacking faecal nitrogen measurements. Consequently, data are based on 63 tanks including 29 duplicate and 5 single tank families

### The relative importance of *EE* and *EG* for *FER*

Of the three covariates and the random family effect in Eq. (2), WTGAIN explained the most variance in *FI* by far, as seen by the 925% increase in its error variance when WTGAIN is omitted from the equation (Results not shown). This was followed in decreasing order of importance by FAMILY (64.2%), EG (55.3%) and W0 (10.9%). The estimated regression coefficient of *FI* shows 0.65 g increase in *FI* per g increase in *WTGAIN.* It also shows a 2.60 g increase in *FI* per 1 kJ/g increase in *EG* (Table 4), which equals to 5.6% of the average feed intake of 46.75 g per fish. In other words, at a given *WTGAIN, FI* increased 5.6% per kJ/g increase in *EG*. The average *EG* was 8.87 kJ/g weight gain, thus according to Eq. (1), if *EF* and *EE* were kept constant, a 1 kJ/g reduction in *EG* from 8.87 to 7.87 kJ/g should give a 11.3% reduction in 1/FER (= FCR). This means an 11.3% reduction in FI at a given WGAIN. However, as the observed effect was half of this (5.6%), fish with reduced body energy (i.e. fat) deposition have higher feed efficiency (FER), but at a cost of reduced energy efficiency (EE).

**Table 4:** Estimates of the effects of the explanatory variables in Eq. (2) on feed intake (g/fish)

Effect	Level	Estimate	Std Error	df^a^	*t*	p-value	Variance component
*Fixed effects*							
*ROOM*^*b*^	1	45.9	0.22	30	-6.46	0.000	
	2	47.1	0.16	30	-6.12	0.000	
*Covariates coeff*							
Initial weight (*W0*), g		0.15	0.041	30	3.73	0.001	
Weight gain (*WTGAIN*), g		0.65	0.018	30	36.79	0.000	
Energy density of gained weight (*EG*), kJ g-1		2.60	0.308	30	8.44	0.000	
*Random effects*							
*FAMILY*							0.419
Error variance							0.616

^a^ Denominator degree of freedom

^b^ Least squares means

**Fig. 3:**
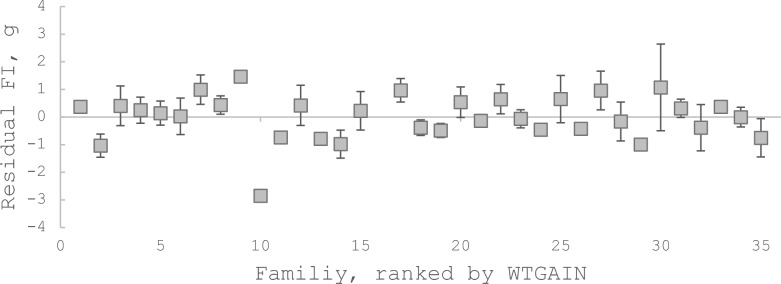
Family mean values for residual feed intake ranked by weight gain. Residual feed intake (RFI; g) calculated from Eq. [2]. N = 34

The variance component for *FAMILY* (Table 4) showed substantial genetic variation in *FI* adjusted for variation in both *W0*, *WTGAIN* and *EG* (i.e. residual feed intake, *RFI*). The estimated heritability for *RFI* calculated based on the estimated variance component from Eq. (3) was, however, low (0.04). This somewhat unexpected result is discussed in the Discussion section. A plot of family mean residual feed intake for the 34 families is given in Fig. 3.

**Fig. 4:**
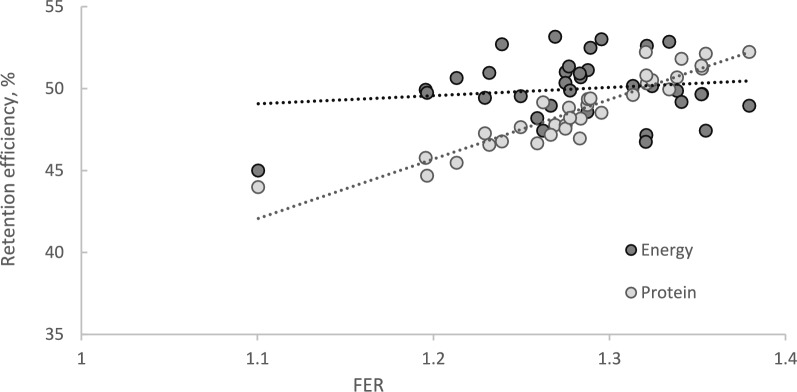
Retention efficiency of energy and protein. Regression of energy efficiency (EE; %) and protein efficiency (PE; %) family means on their feed efficiency ratio (FER). N = 34

As seen in Fig. 4, *FER* of the 34 families was strongly correlated to their protein efficiency (*PE*) (*R*^*2*^ = 0.86,* P* < 0.001). Thus, despite the reduction in energy efficiency (*EE)*, the increased *FER* obtained through reduction in body energy deposition (*EG*) had a positive effect on resource utilisation, through improved PE. The relationship between *FER* and *EE* was, on the other hand, low (*R*^*2*^ = 0.08, *P* < 0.021).

### Genetic analysis

Tables 2 and 3 show that the heritability for intake of energy and crude protein was 0.25 and 0.26, respectively; and those for energy gain and crude protein gain 0.25 and 0.31, respectively. When presented as efficiency traits, i.e. as percentages of feed intake, the heritability estimates for the energy and protein gain were reduced to 0.12 and 0.16, respectively. Thus, selection for the gain traits is expected to be more effective than selection for the efficiency traits. The energy loss by N-excretion had a very low heritability of 0.01.

Table 5 shows that *FI*, *WTGAIN*, *FER* and *EG* had medium heritability, ranging from 0.19 for *FER* to 0.42 for *WTGAIN*. The genetic correlation between *WTGAIN* and *FI* was very high (0.98), while that between *WTGAIN* and *EG* was negative (-0.22).

**Table 5:** Estimates of heritability (on diagonal) and genetic (above diagonal) and residual (below diagonal) correlations (± standard errors) between *FI*, *WTGAIN*, *FER* and *EG*

	*FI*	*WTGAIN*	*FER*	*EG*
*FI*	0.35 ± 0.11	0.98 ± 0.01	0.54 ± 0.16	-0.07 ± 0.20
*WTGAIN*	0.95 ± 0.02	0.42 ± 0.13	0.68 ± 0.12	-0.22 ± 0.19
*FER*	0.02 ± 0.15	0.32 ± 0.13	0.19 ± 0.07	-0.64 ± 0.13
*EG*	0.35 ± 0.13	0.20 ± 0.14	-0.45 ± 0.13	0.26 ± 0.10

*FI* = feed intake; *WTGAIN* = weight gain; *FER* = feed efficiency ratio; *EG* = energy density of gained weight

### Genetic gains

Table 6 shows expected genetic and correlated gains for *WTGAIN, FI*, *EG* and *FER* when the breeding goal include *WTGAIN* (a revenue trait) and *FI* (a cost trait) and selection was practiced for either *WTGAIN* or *WTGAIN* with *FI*, *EG* or *FER*, and with a 1.0 weight on *WTGAIN* and either -0.25, -0.5 or -1.0 for FI. For the intermediate weight on *FI* of -0.5 in the breeding goal, scenario 2 (which included WTGAIN and *FI* in the selection index) and scenario 4 (which included WTGAIN and *FER* in the selection index) had very similar results as scenario 1 (with only *WTGAIN* in the selection index), with rather small changes ± 2% in *WTGAIN* and *FI*, 12–22% increase in *FER* and 37–41% reduction/improvement in *EG*. However, scenario 3 (which included *WTGAIN* and *EG* in the selection index) showed different and positive results of 7% increase in *WTGAIN,* 1% increase in *FI*, 33% increase in *FER* and 163% reduction/improvement in *EG*.

**Table 6:** Genetic gains in WTGAIN and FI and correlated genetic gains in FER and EG (in genetic sd units per unit of selection intensity) for four different selection criterion traits scenario, each with three different sets of weights for the two breeding objective traits WTGAIN and FI

Scenario no	1	2	3	4
Breeding goal	1.0 *WTGAIN*—0.25 *FI*			
Selection index traits	*WTGAIN*	*WTGAIN* *FI*	*WTGAIN* *EG*	*WTGAIN* *FER*
*WTGAIN*	0.633	0.649	0.691	0.651
*FI*	0.632	0.627	0.648	0.629
*FER*	0.436	0.475	0.566	0.485
*EG*	−0.142	−0.184	−0.347	−0.183
Gain in breeding goal	0.475	0.492	0.529	0.494
Breeding goal	1.0 *WTGAIN*—0.5 *FI*			
Selection index traits	*WTGAIN*	*WTGAIN* *FI*	*WTGAIN* *EG*	*WTGAIN* *FER*
*WTGAIN*	0.645	0.646	0.687	0.649
*FI*	0.633	0.620	0.641	0.624
*FER*	0.436	0.487	0.579	0.497
*EG*	−0.142	−0.200	−0.374	−0.195
Gain in breeding goal	0.329	0.336	0.367	0.337
Breeding goal	1.0 *WTGAIN*—1.0 *FI*			
Selection index traits	*WTGAIN*	*WTGAIN* *FI*	*WTGAIN* *EG*	*WTGAIN* *FER*
*WTGAIN*	0.645	0.190	0.339	0.219
*FI*	0.632	0.122	0.258	0.158
*FER*	0.436	0.353	0.480	0.394
*EG*	−0.142	−0.302	−0.566	−0.268
Gain in breeding goal	0.013	0.068	0.081	0.061

*WTGAIN* = weight gain; *FI* = feed intake; *FER* = feed efficiency ratio; *EG* = energy density of gained weight

The gains of the breeding goal for scenarios 2 and 4 were ~ 2.5%, and ~ 11.5% for scenario 3.

When the weights of *FI* in the breeding goal became more extreme (0.25 and 1.0), the tendencies between the gains in the different traits were the same as in the breeding goal with the intermediate weight in the breeding goal. However, the gain in the total breeding goal was almost zero (0.012) with the high weight of -1.0 for *FI*.

Overall, results show that including only *WTGAIN* in the breeding goal result in the lowest total gain and including *EG* in the breeding goal resulted in the highest overall genetic gain for all three breeding goals.

## Discussion

Overall, the results show that selection for *WTGAIN* and against *EG* will improve *FER* without the need of individual *FI* records that are difficult to obtain in fish. The favourable genetic correlations of *FER* with *WTGAIN* (0.68) and *EG* (-0.64) show that both *EG* and *WTGAIN* together provide promising indicator traits for genetic selection for improved feed efficiency, as these traits are both easy to and less costly to measure than feed intake. The magnitude of these genetic correlations and the low genetic correlation between *WTGAIN* and *EG* (-0.22), indicate that a relatively small proportion of the genetic variation in *FER* is caused by variables other than *WTGAIN* and *EG*. Therefore, the potential for obtaining increased genetic gain in *FER* (above that obtained by indirect selection for *WTGAIN* and *EG*) through direct selection for increased *FER* is relatively modest. This confirms earlier studies on rainbow trout reviewed by Knap and Kause [16], but the present study is the first to quantify the partitioning of the saved energy of producing leaner A. salmon into reduced energy efficiency and improved feed (and protein) efficiency.

The analyses of body composition of individual fish confirmed that deposited fat is mainly replacing water, and that body energy is tightly correlated with body fat. The correlation coefficient showed that one g of body fat corresponded to 38.3 kJ of energy. This is slightly lower than the heat of combustion value of 39.5 kJ/g lipid often used in fish nutrition studies (e.g. NRC 1993), and the deviation may be partly explained by the slight reduction in protein with increasing lipid content. Our findings confirm vast literature, e.g. [26], showing that fat is mainly replacing water and that body protein concentration varies little among fish. This supports the contention of Knap and Kause [14] who concluded that although the metabolic efficiency is higher for fat deposition than for protein deposition, e.g. [27], this effect is negligible compared to the effect of replacing body fat with water. The proportionality of lipid, energy and dry matter (or moisture) content in the fish shows that any of these traits, or the lipid:protein ratio [16] can be used to rank fish for body energy content. However, for more detailed studies of energy partitioning, calorimetric data are required.

### Energy partitioning

Since *FER* is proportional to the ratio between *EE* and *EG* (Eq. (1)), reduced fat deposition (i.e. reduced *EG*) will inevitably result in either increased *FER*, reduced *EE*, or a combination of the two. Given constant *EE*, feed intake required to support a given growth rate would be directly proportional to *EG*. Perhaps more likely, some of the reduction in *EG* in leaner families, might have been caused by higher energy losses (e.g. *HEAT*) and thus, reduced *EE*. Our results suggest a “50:50 share” between these two effects in juvenile Atlantic salmon, since the reduction in *FI* at a given growth rate (1/*FER*) obtained by reducing *EG* by 1 kJ/g (11.3% of mean *EG*) was half (5.6%) of that expected if *EE* had been constant (11.3%). To the best of our knowledge, this is the first report quantifying energy and protein partitioning in an elite breeding population of fish, and there are few prior data to compare with. Groot et al. [28] compared energy and nitrogen partitioning of rainbow trout of a fat and a lean strain and found no differences in neither energy efficiency nor feed conversion ratio. Examination of their data reveals that the growth composition (including energy) in the trial period was similar for the two strains, explaining the lack of effects on energy efficiency and feed conversion ratio.

Comparison of retention efficiencies between studies should as far as possible be based on digestible intake, since faecal losses of energy and crude protein depend on feed composition. The mean digestible energy efficiency (*DEE*) and digestible protein efficiency (*DPE*) reported here (61 ± 3 and 55 ± 2%, respectively) are within the ranges of previous reports in Atlantic salmon in freshwater (34–62% and 43–60%; [29–31]) and seawater (40–69% and 42–68%; [7, 32–35]).

Our results show that although improved *FER* with reduced body energy comes with a cost of reduced *EE*, the improvement in *FER* was strongly associated with an improvement in protein efficiency. This was expected since body protein content usually varies only within a narrow range (here, tank mean crude protein was in the range 17.0 – 17.9 kJ/g) and all groups were given the same feed. Low phenotypic correlation between *EE* and *FER*, and a strong correlation between protein efficiency and *FER* (Fig. 4) among Atlantic salmon families, was also seen by Kolstad et al. [3].

Residual feed intake (*RFI*) is a widely used feed efficiency trait in genetic studies with different terrestrial livestock animals and is calculated as the difference between observed and expected FI when taking into account the costs of maintenance and production and many other explanatory variables which are energy sinks (see Martin et al. [36] for a review on the methodology). In fish, *RFI* has been estimated as the residual of a model including fish weight and growth (e.g.* WTGAIN*), but without taking into account the chemical composition of the gain, e.g. [5, 16, 37–40]. Ahmad et al. [41] estimated the heritability of FI phenotypically independent of WTGAIN (pRFI) as 0.11 ± 0.05 – 0.16 ± 0.07.

and FI genetically independent as (gRFI) 0.06 ± 0.03 – 0.18 ± 0.06 for three time points of Atlantic salmon. To test if body composition is an important component of the *RFI*, we omitted *EG* from the model in Eq. (3) and found that EG explained 47% of the variation in *RFI* (*R*^*2*^ = 0.47) among tanks, when *RFI* was calculated from this reduced model. Our original model in Eq. (3), which includes *EG*, is analogous to the one suggested by Knap and Kause [14], using body weight, body weight gain and body composition as independent variables.

Whole body energy gain is linearly correlated to energy intake in salmon [34]. Therefore, *EE* (gain/intake) is increasing with *FI* and *WTGAIN*, since the proportion of the energy intake spent for maintenance is reduced. This is likely to explain the high genetic correlation between *WTGAIN* and *FER*, seen in the present study. When *WTGAIN* and *EG* are accounted for as in the applied model (Eq. (3)), the resulting *RFI* is thought to reflect the biological efficiency at a given intake. *RFI* in terrestrial livestock has been shown to be correlated to energy sinks or explanatory variables e.g. digestibility, maintenance energy requirement, energetic efficiency of mitochondria, feeding behaviour, activity and methane emissions [42–45]. As the (heritable) explanatory variables in the *RFI* model get closer to explaining all the (heritable) variation in feed intake, the (heritability of) unexplained portion (*RFI*) becomes smaller. In the case of land animals, where recording all the different explanatory variables is expensive or infeasible, *RFI* based on the energetic variables that are feasible to record (maintenance, production, growth) is still significantly heritable, it may be optimal to select for *RFI* instead of all the different explanatory energetic phenotypes [46]. In our study, although the variance components (40% family and 60% error) suggested a significant genetic variation, the estimated heritability for *RFI* was very low (0.04 ± 0.03) and not significantly different from zero (although very close to significance). Thus, our results agree with [16] who found that a major part of the potential genetic improvement in feed efficiency in rainbow trout can be realized by selection for increased growth rate and reduced body energy, and that the extra gain that could be obtained by direct selection for *FER* or *RFI*, requiring complex and expensive measurements of *FI*, is limited.

### Breeding goal and selection index

Both *WTGAIN* and *FI* are traits of substantial economic importance and thus obvious breeding goal traits. However, *FI* is difficult to measure and therefore also to directly select for. Also, all ratio traits are difficult to improve genetically because the change due to selection will be non-linear for the two traits in the ratio, and an animal with a good *FER* may either grow poorly but have low feed intake or grow well but may also eat a lot. The latter is clearly more profitable. A linear increase is not expected, e.g. because of the different heritability of the two traits and genetic correlations being less than 1.0 [47] (although it was very high here at 0.98); *EG* had a genetic correlation to *FER* of -0.64. These results confirm earlier results by [48] that estimated genetic correlation between weight gain and fillet fat of − 0.45 ± 0.17 for fish slaughtered at about the same body weight. This is almost the same magnitude as the genetic correlation between *WTGAIN* and *FER* (0.68). The genetic correlation between *EG* and *WTGAIN* was only -0.22, showing that they are indeed rather different traits genetically. *WTGAIN* has traditionally been very successful in improving *FER* due to the favourable genetic correlation [7]. The genetic analysis of this experiment confirms that *EG* is a valuable trait to include in a selection index for Atlantic salmon. When adding *EG* to the selection index in addition to *WTGAIN*, all the analysed traits (*WTGAIN, FI, FER* and* EG*) were improved, and genetic gain of the breeding goal increased 11.6%. The two breeding goal traits *WTGAIN* and *FI* increased 6.5 and 1.3%, respectively. These results confirm the conclusion of Knap and Kause [14].

The significant positive phenotypic correlation between growth rate and feed efficiency has also been reported amongst family groups of Atlantic salmon [3], although the number of families in that study was too low to estimate genetic correlations. The main biological explanation for this positive correlation is that fast production reduces the proportion of energy intake used for maintenance. The genetic correlation between *WTGAIN* and *FI* was very high (0.98). Using the X-ray method of feed intake recording on sibs of the fish used here, Difford et al. [49] reported a genetic correlation of 0.81 ± 0.09 between individual feed intake and average daily weight gain. This shows that in these data sets there is very little room for genetic improvement of residual feed intake, i.e. feed intake not related to *WTGAIN*, although small improvements of this trait may yield high economic benefits as feed account for a large proportion of the total cost [1]. However, by further partitioning *WTGAIN* into more precise compositional phenotypes, it is possible to obtain correlated genetic gains in *FER* without the inclusion of *FI* itself. Importantly, precise, long-term measurements of feed intake are necessary for direct selection for reduced *FI* and increased *FER* and for understanding the relative importance of energy partitioning phenotypes. The fish used here were however only tested during a relative short period (seven weeks) in the freshwater phase, and most likely the results might not be directly transferred to the seawater phase, which is where most of the growth takes place. Given the promising findings herein that selection for increased growth and reduced body energy is adequate to capture a very large proportion of the genetic variation in feed efficiency without FI records, similar studies for Atlantic salmon in the sea water phase are highly needed.

### Methods

The chosen experimental design, which only recorded tank mean data (except for *WTGAIN*), proved useful to estimate genetic parameters for the four traits when a genomic analysis was used with SNP array data of ~ 50000 SNPs. Although genetic parameters are expected to be estimated without bias, the use of tank-means implies that tank residuals, i.e. the residual of Eq. (3), cannot be split into environmental effects of the tank and the individual (within tank) environmental effects. This affects the estimates of heritability as shown below.

The heritability of *FI*, *WTGAIN, FER* and *EG* were moderate; *FER* had lowest heritability (0.16). The environmental variances (*s*_*e*_^*2*^), which are needed for the denominator in the calculation of heritability, where assumed here to equal *s*_*e*_^*2*^ = 25*s*_*r*_^*2*^, which assumes that the variance due to tank effects is 0, i.e. environmental effects between the same trait in replicated tanks are uncorrelated. In the presence of tank effects, the environmental effects show an intra-class correlation of *t*, and the environmental variance is *s*_*e*_^*2*^ = *s*_*r*_^*2*^/[*t* + (1-*t*)/25], which varies between 25*s*_*r*_^*2*^ and 1*s*_*r*_^*2*^, for *t* = 0 to *t* = 1, respectively, where *t* = 1 (no individual environmental variance) is very unrealistic. Hence, we may have used an over-estimate of the environmental variance for the heritability estimation, which implies that the heritability in Tables 2, 3 and 5 are conservative estimates.

Fat and energy content of the fish can be accurately analysed by standard laboratory methods, but this requires killing the fish before analyses. For effective selection of breeding candidates, non-destructive methods would be preferable. Estimation of fat content in whole or live salmon has been done by different spectroscopic methods [10–13]. These studies have mainly been aiming on measuring in fillets, using muscle samples as reference for calibration. Although muscle is the major fat storage tissue, accounting for around 60% of the fat stores in harvest sized salmon (calculated from [19]), better calibration against body fat is needed. Selection against visceral fat, instead of muscular fat, would be preferable since this might also increase the edible yield [50]. However, since muscle is such a dominant lipid storage tissue in salmon, reduction in both is probably necessary to increase the gain in feed efficiency.

The choice of working with small fish in small units gave us the possibility to measure exact feed intake, feed efficiency and energy partitioning in a relatively large number of replicated family groups, which would not be affordable with larger fish. The major drawbacks with our approach are the questionable transferability of the results to large fish in seawater [51] and the lack of individual phenotyping of traits other than fish weights and growth rate. Phenotyping feed intake in individual fish is costly and requires treatments that may be stressful to the fish and affect the measured intake, such as solitary housing [5] or repeated X-raying, e.g. [4, 49, 52]. With existing methods, the relatively precise estimates on energy partitioning presented here could only be obtained on a group (tank) level.

## Conclusions

Genetic selection for weight gain and against body energy will improve feed efficiency ratio without the need of individual feed intake records in Atlantic salmon. The results suggest that by selection against body energy, and given the same weight gain, 50% of the reduction in body energy can be realised as reduced feed intake and thus improved FER.

## Data Availability

The datasets generated and/or analysed during the current study are not publicly available due to commercial interests but are available on reasonable request.

## References

[1] Iversen A, Asche F, Hermansen Ø, Nystøyl R. Production cost and competitiveness in major salmon farming countries 2003–2018. Aquaculture. 2020;522:735089.

[2] Ziegler F, Jafarzadeh S, Skontorp Hognes E, Winther U. Greenhouse gas emissions of Norwegian seafoods: from comprehensive to simplified assessment. J Ind Ecol. 2022;26:1908–19.

[3] Kolstad K, Grisdale-Helland B, Gjerde B. Family differences in feed efficiency in Atlantic salmon (*Salmo salar*). Aquaculture. 2004;241:169–77.

[4] Dvergedal H, Ødegård J, Øverland M, Mydland LT, Klemetsdal G. Selection for feed efficiency in Atlantic salmon using individual indicator traits based on stable isotope profiling. Genet Sel Evol. 2019;51:13. 30991944 10.1186/s12711-019-0455-9 PMC6466720

[5] de Verdal H, Komen H, Quillet E, Chatain B, Allal F, Benzie JA, et al. Improving feed efficiency in fish using selective breeding: a review. Rev Aquac. 2018;10:833–51.

[6] Jobling M, Covs D, Damsgard B, Kristiansen H R, Koskela J, Petursdottir TE, et al. Techniques for measuring feed intake. In: Houlihan D, Boujard T, Jobling M, editors. Food intake in fish. Wiley Online; 2007. p. 49–87.

[7] Thodesen J, Grisdale-Helland B, Helland SJ, Gjerde B. Feed intake, growth and feed utilization of offspring from wild and selected Atlantic salmon (*Salmo salar*). Aquaculture. 1999;180:237–46.

[8] Einen O, Holmefjord I, Åsgård T, Talbot C. Auditing nutrient discharges from fish farms: theoretical and practical considerations. Aquac Res. 1995;26:701–13.

[9] Kent M, Lees A, Christie RH. Seasonal variation in the calibration of a microwave fat: water content meter for fish flesh. Int J Food Sci Technol. 1992;27:137–43.

[10] Wold JP, Isaksson T. Non‐destructive determination of fat and moisture in whole Atlantic salmon by near‐infrared diffuse spectroscopy. J Food Sci. 1997;62:734–6.

[11] Solberg C, Saugen E, Swenson LP, Bruun L, Isaksson T. Determination of fat in live farmed Atlantic salmon using non‐invasive NIR techniques. J Sci of Food Agric. 2003;83:692–6.

[12] Veliyulin E, van der Zwaag C, Burk W, Erikson U. In vivo determination of fat content in Atlantic salmon (*Salmo salar*) with a mobile NMR spectrometer. J Sci Food Agric. 2005;85:1299–304.

[13] Folkestad A, Wold JP, Rørvik KA, Tschudi J, Haugholt KH, Kolstad K, Mørkøre T. Rapid and non-invasive measurements of fat and pigment concentrations in live and slaughtered Atlantic salmon (*Salmo salar L.*). Aquaculture. 2008;280:129–35.

[14] Knap P, Kause A. Phenotyping for genetic improvement of feed efficiency in fish: lessons from pig breeding. Front Genet. 2018;9:184. 29881397 10.3389/fgene.2018.00184 PMC5976999

[15] Janhunen M, Nousiainen A, Koskinen H, Vehviläinen H, Kause A. Selection strategies for controlling muscle lipid content recorded with a non-destructive method in European whitefish, *Coregonus lavaretus*. Aquaculture. 2017;481:229–38.

[16] Kause A, Kiessling A, Martin SAM, Houlihan D, Ruohonen K. Genetic improvement of feed conversion ratio via indirect selection against lipid deposition in farmed rainbow trout (*Oncorhynchus mykiss Walbaum*). Br J Nutr. 2016;116:1656–65. 27813470 10.1017/S0007114516003603

[17] Quinton C, Kause A, Ruohonen K, Koskela J. Genetic relationships of body composition and feed utilization traits in European whitefish (*Coregonus lavaretus L*.) and implications for selective breeding In. fishmeal- and soybean meal­ based diet environments. J Anim Sci. 2007;85:3198–208. 10.2527/jas.2006-792 17709787

[18] Aas TS, Ytrestøyl, T Åsgård, T. Utilization of feed resources in the production of Atlantic salmon (*Salmo salar*) in Norway: An update for 2016. Aquac Rep. 2019;15:100216.

[19] Aas TS, Åsgård T, Ytrestøyl T. Chemical composition of whole body and fillet of slaughter sized Atlantic salmon (*Salmo salar*) and rainbow trout (*Oncorhynchus mykiss*) farmed in Norway in 2020. Aquac Rep. 2022;25:101252.

[20] Helland SJ, Grisdale‐Helland B, Nerland S. A simple method for the measurement of daily feed intake of groups of fish in tanks. Aquaculture. 1996;139:157–63.

[21] Austreng E. Digestibility determination in fish using chromic oxide marking and analysis of contents from different segments of the gastrointestinal tract. Aquaculture. 1978;13:265–72.

[22] Elliott JM, Davison W. Energy equivalents of oxygen consumption in animal energetics. Oecologia. 1975;19:195–201. 28309234 10.1007/BF00345305

[23] Forsberg OI. The impact of varying feeding regimes on oxygen consumption and excretion of carbon dioxide and nitrogen in post‐smolt Atlantic salmon (*Salmo salar L*.). Aquac Res. 1997;28:29–41.

[24] Gilmour AR, Gogel BJ, Cullis BR, Welham SJ, Thompson R. ASReml User Guide Release 4.1 Functional Specification. 2015. www.vsni.co.uk. https://www.hpc.iastate.edu/sites/default/files/uploads/ASREML/UserGuideFunctional.pdf. Accessed 14 Janv 2026.

[25] Neter J, Kutner MH, Nachtsheim CJ, Wasserman W. Applied Linear Statistical Models. 4th ed. New York: WCB McGraw-Hill; 2016.

[26] Shearer KD, Åsgård T, Andorsdottir G, Aas GH. Whole body elemental and proximate composition of Atlantic salmon (*Salmo salar*) during the life cycle. J Fish Biol. 1994;44:785–97.

[27] Emmans GC. Effective energy: a concept of energy utilization applied across species. Br J Nutr. 1994;71:801–21. 8031731 10.1079/bjn19940188

[28] Groot R, Lyons P, Schrama JW. Differences in energy utilisation between a lean and fat strain of rainbow trout (*Oncorhynchus mykiss*). Aquaculture. 2022;561:738681.

[29] Grisdale-Helland B, Helland SJ. Replacement of protein by fat and carbohydrate in diets for Atlantic salmon (*Salmo salar*) at the end of the freshwater stage. Aquaculture. 1997;152:167–80.

[30] Thodesen J, Gjerde B, Grisdale-Helland B, Storebakken T. Genetic variation in feed intake, growth and feed utilization in Atlantic salmon (*Salmo salar*). Aquaculture. 2001;194:273–81.

[31] Azevedo PA, Leeson S, Cho CY, Bureau DP. Growth and feed utilization of large size rainbow trout (*Oncorhynchus mykiss*) and Atlantic salmon (*Salmo salar*) reared in freshwater: diet and species effects, and responses over time. Aquac Nutr. 2004;10:401–11.

[32] Refstie S, Olli JJ, Standal H. Feed intake, growth, and protein utilisation by post-smolt Atlantic salmon (*Salmo salar*) in response to graded levels of fish protein hydrolysate in the diet. Aquaculture. 2004;239:331–49.

[33] Aas TS, Grisdale-Helland B, Terjesen BF, Helland SJ. Improved growth and nutrient utilisation in Atlantic salmon (*Salmo salar*) fed diets containing a bacterial protein meal. Aquaculture. 2006;259:365–76.

[34] Helland SJ, Hatlen B, Grisdale-Helland B. Energy, protein and amino acid requirements for maintenance and efficiency of utilization for growth of Atlantic salmon post-smolts determined using increasing ration levels. Aquaculture. 2010;305:150–8.

[35] Liu B, Liu Y, Liu Z, Qiu D, Sun G, Li X. Influence of stocking density on growth, body composition and energy budget of Atlantic salmon (*Salmo salar L.*) in recirculating aquaculture systems. Chin J of Oceanol Limnol. 2014;32:982–90.

[36] Martin P, Ducrocq V, Faverdin P, Friggens NC. Disentangling residual feed intake—insights and approaches to make it more fit for purpose in the modern context. J Dairy Sci. 2021;104:6329–42. 33773796 10.3168/jds.2020-19844

[37] Doupe RG, Lymbery AJ. Indicators of genetic variation for feed conversion efficiency in black bream. Aquac Res. 2004;35:1305–9.

[38] Martins CI, Hillen B, Schrama JW, Verreth JA. A brief note on the relationship between residual feed intake and aggression behaviour in juveniles of African catfish (*Clarias gariepinus*). Appl Anim Behav Sci. 2008;111:408–13.

[39] Daulé S, Vandeputte M, Vergnet A, Guinand B, Grima L, Chatain B. Effect of selection for fasting tolerance on feed intake, growth and feed efficiency in the European sea bass (*Dicentrarchus labrax*). Aquaculture. 2022;420:42–9.

[40] Gomez-Raya L, Rauw WM, Cabaleiro S, Caamaño R, Garcia-Cortes LA, Kause A. The relationship between feed efficiency, growth and group dominance dynamics in turbot (*Scophthalmus maximus*). Span J Agric Res. 2018;16:e0604.

[41] Ahmad A, Sonesson AK, Hatlen B, Bæverfjord G, Berg P, Norris A, et al. Genetic analysis of individual feed intake and efficiency in Atlantic salmon smolts using X-ray imaging. Aquaculture. 2025;608:742715.

[42] Herd RM, Arthur PF. Physiological basis for residual feed intake. J Anim Sci. 2009;87:e64-71. 19028857 10.2527/jas.2008-1345

[43] Young JM, Dekkers JCM. The genetic and biological basis of residual feed intake as a measure of feed efficiency. In: Patience JF, editor. Feed efficiency in swine. Wageningen: Wageningen Academic Publishers; 2012. p. 153–66.

[44] Løvendahl P, Difford GF, Li B, Chagunda MGG, Huhtanen P, Lidauer MH, et al. Selecting for improved feed efficiency and reduced methane emissions in dairy cattle. Animal. 2018;12:336–49. 10.1017/S1751731118002276 30255826

[45] Manzanilla-Pech CIV, Difford GF, Løvendahl P, Stephansen RB, Lassen J. Genetic (co-) variation of methane emissions, efficiency, and production traits in Danish Holstein cattle along and across lactations. J Dairy Sci. 2022;105:9799–809. 36241442 10.3168/jds.2022-22121

[46] Stephansen RB, Martin P, Manzanilla-Pech CIV, Gredler-Grandl B, Sahana G, Madsen P, et al. Novel genetic parameters for genetic residual feed intake in dairy cattle using time series data from multiple parities and countries in North America and Europe. J Dairy Sci. 2023;106:9078–94. 37678762 10.3168/jds.2023-23330

[47] Sutherland TM. The correlation between feed efficiency and rate of gain, a ratio and its denominator. Biometrics. 1965;21:739–49. 5858102

[48] Kristjánsson OH, Gjerde B, Ødegård J, Lillehammer M. Quantitative genetics of growth rate and filet quality traits in Atlantic salmon inferred from a longitudinal Bayesian model for the left-censored Gaussian trait growth rate. Front Genet. 2020;11:1–15. 33329713 10.3389/fgene.2020.573265 PMC7734147

[49] Difford GF, Hatlen B, Gjerde B, Heia K, Baeverfjord G, Norris A, et al. Validation and genetic parameters of the X-ray method for phenotyping individual feed intake in Atlantic salmon. Aquaculture. 2024;586:740738.

[50] Einen O, Waagan B, Thomassen MS. Starvation prior to slaughter in Atlantic salmon (*Salmo salar*): I. Effects on weight loss, body shape, slaughter-and fillet-yield, proximate and fatty acid composition. Aquaculture. 1998;166:85–104.

[51] Ahmad A, Wold JP, Sonesson AK, Hatlen B, Dagnachew BS, Berg P, et al. Genetic and phenotypic validation of whole body fat content measured across production phases of Atlantic salmon using dielectric and near infrared interactance spectroscopy. Aquaculture. 2025;596:741747.

[52] Kause A, Tobin D, Houlihan DF, Martin SAM, Mäntysaari EA, Ritola O, et al. Feed efficiency of rainbow trout can be improved through selection: different genetic potential on alternative diets. J Anim Sci. 2006;84:807–17. 16543557 10.2527/2006.844807x

